# Supplementary material for: Are carbon emissions trading and green financial instruments synergistic? -Comprehensive quantitative research based on content analysis
Source: PLoS One. 2024 Mar 7;19(3):e0298601. doi: 10.1371/journal.pone.0298601 (PMC10919601; doi:10.1371/journal.pone.0298601)
Supplement: S1 Data — (ZIP) [file pone.0298601.s001.zip › Data/地级市Z_F工作报告.zip!/╣π╢1⁄2/╟σ╘╢╩╨/└┤╫╘╣1⁄2╓┌║┼í╛┬φ┐╦╩2╛▌═°í┐_2017.docx]

各位代表：

我代表市人民政府向大会作工作报告，请予审议，并请政协各位委员和其他列席人员提出意见。

本届政府工作回顾

2016年是本届政府任期最后一年，也是“十三五”的开局之年。一年来，在省委、省政府和市委的正确领导下，在市人大和市政协的监督支持下，市政府全面贯彻党的十八大、十八届三中、四中、五中、六中全会和习近平总书记系列重要讲话精神，真抓实干，攻坚克难，经济社会持续健康向好，基本完成市六届人大七次会议确定的目标任务，实现了“十三五”的良好开局。

——经济发展稳步增长。预计全市完成生产总值1375亿元，增长8%；社会消费品零售总额621亿元，增长9%；外贸进出口总额283.1亿元，增长1.2%；规上工业增加值448亿元，增长9.8%；完成固定资产投资629.9亿元，增长1.5%。受营改增、降成本和土地出让等因素影响，一般公共预算收入95.63亿元，可比口径负增长7.76%。金融机构本外币存款、贷款余额分别增长11.6%和7%。新登记各类市场主体3.38万户，同比增长11%，其中企业增长23%。接待旅游3493.7万人次，实现旅游收入269亿元，分别增长5.3%、11.6%。

——“南融北康”全面实施。省正式印发广清一体化“十三五”发展规划。20亿元的穗清基金正式落地。广清产业园清城片区累计签约项目104个，44家企业动工，10家企业投产；佛冈片区开发正式启动。完成广清一体化交通专项规划编制，省道114线广清产业园至花都段改造完成。185所学校、15家县级以上医院与广州建立帮扶关系。连阳四县（市）纳入国家重点生态功能区，每年每县可获9000万元左右的中央财政专项补助。编制连阳地区旅游发展总体规划，制定“三连一阳”联合招商方案，合作共建民族工业园，推进北部地区抱团发展。广连高速、二广与清连高速连接线前期工作完成，连州、英德通用机场预选址确定，柳韶铁路列入国家“十三五”规划。

——重大项目加快推进。广清高速扩建工程全线通车。广清城轨清远段首期全面贯通。北江五桥主体合拢。长隆种源基地首期、华南虎野化训练基地等项目建成，主体工程及酒店群正式动工，已投放动物50种1000多头（只）。神华国华一、二期完成五通一平。通用电梯首期征地完成。汕昆、汕湛等5条在建高速公路、北江航道扩能升级工程有序推进。省级职教基地二期前期工作基本完成。抽水蓄能、金发一期、万达广场等重大项目建成投产。省、市重点项目完成投资390.3亿元，完成年度投资计划的104.8%。

——城乡面貌日新月异。全力推进创文、创卫突出问题综合整治，新建、改造提升37个农贸市场，整治、规范沿街店铺招牌1.1万多处，拆除各类乱搭乱建、违章建筑1970多处，修复路面17.5万平方米，整治河道25条（段），新划停车泊位5万个，“奋战四个月，改变一座城”目标基本实现，城市环境明显改善，讲文明、树新风氛围日益浓厚。创卫工作通过国家明查暗访。中心城区新启动项目61个，凤翔南路、东门塘环境综合整治等28个项目完工或基本完工。各县城中心镇完成一批城市提质项目。新建美丽乡村142条。英德、连州、阳山三个省级新农村连片示范建设进展顺利。

——综合改革成效明显。加快推进供给侧结构性改革，商品房销售面积831.2万平方米，增长42%，消化库存236.8万平方米，去化周期缩短11.5个月；为企业减负40.28亿元；化解落后钢铁产能62万吨；出清国有“僵尸企业”14家，农网、水利等补短板工作加快。农村综合改革不断深入，共成立村民理事会16464个、经济合作社19569个，累计整合耕地面积106.5万亩，整合涉农资金16.9亿元，建成涉农平台260个、信用合作部49家。成为全国涉农资金整合优化唯一地级试点市，“三个重心下移”模式入选“2016年全国十佳创新社会治理案例”。连续两年获评“全国社会治理创新优秀城市”。相对集中行政许可权试点、“一门式一网式”改革有序推进。在粤东西北率先推进企业登记“七证合一”。

——转型创新取得实效。清远高新区新增国家火炬高性能结构材料特色产业基地、国家科技服务业区域试点等多块“国字号”牌子。华炬科技企业孵化器成为粤东西北首个国家级孵化器，天安智谷智汇空间成为国家级众创空间。乌克兰新材料产品展示中心落户我市。成功承办全国新材料高峰论坛、广东年度经济风云榜颁奖典礼、广佛莞清港澳台七地经济论坛等重要经济活动。天安智谷园区及广东天农集团荣登本届广东年度经济风云榜。推进质量强市建设，我市企业参与制定22项国家标准、3项行业标准。高新技术企业达112家。专利申请量、发明专利申请量分别增长123.2%、100.3%，专利增长连续两年全省第一。工业技改投资增长50%。全省首个新能源电动车产业项目落户英德。富强、雅迪等一批投资超亿元的项目建成投产。

——环境质量持续改善。在全省地级市中率先实行环保考核第三方评价机制。出台了我市首部地方法规《清远市饮用水源水质保护条例》。首次划定各县市黄标车限行区。省挂牌督办的电子废弃物污染环境问题实现摘牌。淘汰黄标车9933辆。推进黄坑河、澜水河等4条黑臭水体整治。26项“创模”指标有22项达标或基本达标。二氧化硫、一氧化碳浓度比上年分别下降22.1%、11.1%。北江干流水质良好，主要江河国控、省控监测断面全部达标。

——民生实事全面完成。全市民生投入237.5亿元，占一般公共预算支出78.3%。新增城镇就业4.6万人。底线民生保障标准进一步提高。建设保障性安居房5937套（户）。建设开放运动场馆109个。改造建设农村危房12151套。完成内街小巷改造100条。新建绿道19公里。解决10万名农村居民自来水供应问题。完成14间乡镇卫生院业务用房扩建任务。为全市全科医生配备一体式巡诊箱。为150个村卫生站配备网络版十二道心电图机。为1.6万农村妇女开展乳腺癌、宫颈癌免费筛查。启动卫生强市、教育现代化先进市创建工作。成功举办第十届中国民间艺术节、2016全国广电公益广告论坛等重大文化活动。全国广电公益广告研创基地落户我市。食品重点品种溯源覆盖率100%。各类劳资纠纷案件下降57.6%。新时期精准扶贫三年攻坚扎实推进，完成4.5万相对贫困人口脱贫任务。

各位代表，2016年各项工作取得了扎实成效，标志着本届政府各项目标任务的圆满完成。五年来，面对复杂严峻的经济形势和改革发展稳定的艰巨任务，我们抢抓省促进粤东西北振兴发展、广州对口帮扶等重大历史机遇，主动适应经济发展新常态，着力打基础谋长远，坚定不移推动转型振兴发展，在全面建成小康社会的道路上迈出了坚实步伐。

过去五年，是奠定我市振兴发展基石的五年。生产总值、财政总收入、社会消费品零售总额、规上工业增加值分别增长43.3%、29.5%、42.2%和29.6%。固定资产投资是上个五年的1.5倍。特别是，广清一体化发展上升到省级战略层面，发展优势得到市场更加广泛认可。清远长隆、神华国华、金发科技等重大项目实现突破，产业集聚和结构调整的动力更加强劲。清远国家高新区、广清产业园、华侨工业园、“两德”合作区、燕湖新城、省级职教基地等重大平台加快建设，“五纵三横、一江三轨”的交通网络初具规模，加快发展的基础和条件更加突出。行政审批、商事登记、投融资体制等多项改革走在粤东西北前列，洼地效应更加凸显。主体功能区建设和农综改探索不断深入，绿色生态发展的路子更加清晰。

过去五年，是转型发展迈出坚实步伐的五年。实施工业转型攻坚，推动陶瓷、水泥、再生金属等产业转型提升。工业技改投资年均增长45%，高耗能行业贡献率下降18.7个百分点，单位GDP能耗下降22.2个百分点。新材料、汽车配件、生物医药等新的主导产业加快形成，现代金融、物流、旅游等服务业成为拉动经济增长的重要力量。高技术制造业、先进制造业增加值年均分别增长26.6%、31.2%，第三产业占GDP比重提高5.2个百分点，新增各类金融机构29家、4A级以上景区7个，建成电商产业园5个。大力推进创新驱动发展，加快建设粤北创新高地。清远高新区成为粤东西北第二个国家级高新区，在全省率先实施科技创新券，天安智谷、华南863、华大电商、融合创富等一批“双创”平台加快建设，高新技术企业数居粤东西北第2位。人才工作取得扎实成效，建成省级以上工程技术研发中心33家，6家企业在新三板挂牌，80家企业在区域股权交易中心挂牌。园区提质增效明显。建成4个省级产业园、6个产业集聚区。新引进工业项目710个。园区平台规上工业增加值、税收占全市比重分别提高18.1和14.8个百分点。

过去五年，是区域协调发展明显加快的五年。深入实施“桥头堡”战略。建立完善广清对口帮扶工作机制，共实施帮扶和一体化项目823个，广清对口帮扶省考核位居第一，广清交通互联、产业互补、生态共建、社会共融的态势初步形成。积极融入广佛肇清云韶经济圈。不断深化与郴州、永州、贺州等周边城市的交流合作。大力推进主体功能区建设试点，建立财政、产业、生态补偿等配套政策，取消对连山、连南、阳山GDP增速、工业发展的考核，推动差异化发展。民族地区扶持力度不断加大，市财政下达连山、连南转移支付资金年均增长39%。北部地区发展条件不断改善，绿色生态发展步伐加快，打造了一批特色鲜明的农林产品和旅游产品，风电、光伏、水电等绿色能源产业快速发展，矿产品深加工产业初具规模。连州、阳山成为国家级出口食品农产品质量安全示范区，连山创建为全国有机农业（水稻）示范基地，连南列入国家全域旅游示范区创建名单，阳山成为国家首批绿色能源示范县。

过去五年，是城乡环境发生显著变化的五年。实施交通大会战，城轨、高速、航道、国省道、县乡公路建设全面推进，积极谋划机场、高铁、轨道交通，完成交通基础设施投资392.5亿元，是上个五年的1.7倍。新建成二广、乐广两条高速公路。完成998公里国省道大修，全面消灭市域内国省道烂路。全市高速公路总里程居全省第2位，等级公路总里程居全省第1位。“一心两核”城市建设全面铺开。燕湖新城完成投资140亿元，骨干框架基本建成。清新加快融入大市区。市中心区域面积扩大15平方公里。推进“五城同创”，成为国家园林城市，城市功能环境大幅提升。英德、连州城市集聚能力不断增强，各县城面貌日新月异。特色小镇建设和镇村规划编制有序铺开。全市建成绿道719.1公里，新增变电容量419万千伏安，光纤入户率34.1%，4G网络实现城乡全覆盖，治理中小河流1300公里，建成美丽乡村419条。

过去五年，是生态综合竞争力不断增强的五年。以前所未有的力度推进环境治理，成立市环境保护委员会，成为全省第三个、粤东西北首个成立“环保警察”的地级市，全力推进“创模”攻坚。市区瓷土全部集中配送，龙塘、石角园区外拆解散户全面取缔，淘汰黄标车5.4万辆，空气质量逐年好转。北江流域保护治理得到加强。新建、改建污水处理厂6家，新增污水处理能力16.5万吨/日，整治规模化禽畜养殖场1629家，完成大燕河、澜水河等一批整治工程，北江流域跨界水质每年100%达标，饮用水质保持优良。完成重金属生态修复首期工程，实施复绿治理矿山48个，城镇生活垃圾无害化处理率91.2%，农村生活垃圾清运处理工作全面铺开。全面推进新一轮绿化广东大行动，完成造林面积198.4万亩、重点碳汇工程108.7万亩、生态景观林带902.6公里、森林公园60个、湿地公园10个；森林覆盖率72%，提高3.6个百分点。

过去五年，是“三农”发展取得有效突破的五年。推动以“三个重心下移”、“三个整合”为重点的农村综合改革，成为全国农村改革试验区，涌现出熊屋村、叶屋村、元江村、新城村等一批典型，基层改革探索经验部分转化为中央决策，农村发展内生动力不断增强。全市农业总产值年均增长4.8%，增速居全省第1位；农民人均可支配收入年均增长12.8%，高于GDP增速5.3个百分点。大力实施“三品”工程，加快培育新型经营主体，形成茶业、草食动物、北江特色渔业、花卉苗木等一批特色农业支柱产业，新增农民专业合作社4104家、家庭农场880个、省级农业龙头企业15家，“三品一标”农产品256个。完成高标准农田建设143.1万亩。农村金融服务体系不断完善。农村电商服务体系全省覆盖面最广。乡村旅游、休闲农业蓬勃发展。

过去五年，是人民群众得到更多实惠的五年。民生累计投入737亿元，年均增长22%，占一般公共预算支出比重提高11.9个百分点。居民人均可支配收入增长57.4%，高于同期GDP增幅14.1个百分点。城镇登记失业率控制在3.5%以内。基本公共服务均等化水平大幅提升，2015年综合排名全省第7位，基本实现“两个80%”的目标。成为粤东西北首批省教育强市，实现全国义务教育发展基本均衡县域全覆盖，教学质量跃居粤东西北前列。新建和改扩建中小学校165所，新增学位7.07万个，扭转多年学生净流出局面。就业服务网络实现100%村居全覆盖。实现在全省294家医院异地就医医保即时结算，大病补充医保工作走在全省前列。县级公立医院改革实现全覆盖，在全省率先实施乡镇卫生院“五个一”工程，村卫生站规范化建设完成率94.7%，市域内住院率达90.5%，群众“看病难、看病贵”问题有效缓解。成功举办清远国际马拉松赛、国际公路自行车赛等系列赛事活动，连州国际摄影展、瑶壮民族文化、“北江舞台、开心周末”等本土文化品牌进一步擦亮。全面完成两轮扶贫开发“双到”任务，国家扶贫试验区建设扎实推进。完成90项民生实事，解决一批事关群众切身利益的热点难点问题。

过去五年，是政府自身建设全面加强的五年。主动接受人大、政协监督，五年共办理415件人大代表建议、1148件政协提案，办复率100%。着力打造“审批环节最少、效率最高、服务最优”的新型城市，在粤东西北率先启动商事制度改革，率先出台权责清单、后续监管清单和企业投资负面清单，网上办事大厅建设水平走在全省前列。市一级行政审批事项调整比率达57%，审批承诺时限压缩58.5%。“两建”平台、12345政府服务热线平台建成使用。完成市、县两级政府机构改革。不断完善政府重大行政决策程序，发挥政府顾问作用，提升决策水平。建立政府法律顾问制度，法治政府建设成效明显。严格落实党风廉政建设责任制，强化行政监察和审计监督，反腐倡廉工作取得实效。认真学习贯彻习近平总书记系列重要讲话精神，严格执行中央八项规定，扎实开展群众路线教育实践、“三严三实”、“两学一做”等学习教育，持续开展“正风”行动和“公述民评”活动，机关作风明显改善。扎实推进“平安清远”创建、食品药品监管和安全生产工作，推进社区治理改革，抓好信访维稳工作，确保了社会大局稳定。国防动员、人民武装、民族、宗教、外事、侨务、港澳、对台、妇女儿童、老龄、残疾人、红十字、档案、人防、方志、气象等工作取得新成绩。

各位代表，五年的成绩来之不易，这是省委、省政府和市委正确领导的结果，是历届班子承前启后、继往开来、共同努力的结果，是全市人民辛勤劳动、团结拼搏的结果，是广州、佛山等兄弟城市及社会各界大力支持的结果。在此，我谨代表清远市人民政府，向全市人民、驻清解放军和武警部队官兵、各民主党派、人民团体以及社会各界人士表示崇高的敬意！向所有关心支持清远发展的港澳台同胞、海外侨胞和国际友人表示衷心的感谢！

回顾过去五年，我们深深体会到，做好政府工作，推动清远又好又快发展，必须牢固树立创新、协调、绿色、开放、共享发展理念，把握好科学发展的方向，锲而不舍推动转型发展和振兴发展；必须把握规律，因地制宜，坚持系统谋划与分类指导相结合，走差异化发展新路；必须坚持深化改革，增创体制机制新优势，为清远发展注入源源不断活力；必须坚持以人民为中心，尊重民意，把民生幸福作为最高追求，着力增进民生福祉；必须坚持民主科学决策，集思广益，群策群力，依法行政，廉洁高效，全面提高机关队伍素质和行政效能。

我们也清醒地认识到，我市经济社会发展还存在不少困难和问题：经济总量不大、质量不高，税源不足，财政收支压力较大；产业结构还需进一步优化，传统高耗能产业占比仍然偏高，新的支柱产业还没有形成，供给侧结构性矛盾比较突出；园区实体经济发展滞后，工业投资不足，部分园区落地项目不多；新型城镇化发展水平不高，产城融合不够，人口集聚力不强；土地、环境、节能减排等要素制约日益趋紧，转型发展更加迫切；区域发展不平衡，南北、城乡差距仍然较大，基本公共服务均等化水平还不高，民生保障水平有待提升；政府行政效能和社会管理水平仍需不断提高，等等。对于这些问题，我们必须高度重视，采取更加有力的措施认真研究解决。

新一届政府目标任务

今后五年，是清远加快振兴发展、全面建成小康社会的攻坚期。综合研判，我们仍处于大有可为的重要战略机遇期，同时也面临许多困难和挑战。全球经济逐步复苏，国际分工格局加快调整，新产业新业态不断涌现；国家“一带一路”、“中国制造2025”、创新驱动等战略全面实施，供给侧结构性改革深入推进，新型城镇化步伐加快，实体经济发展加大力度，国内经济长期向好的基本面没有改变；省大力推进珠三角与粤东西北一体化发展，产业共建力度空前；我市扎实打基础、促转型，交通、城市、园区等基础条件有效改善，一批重大产业项目动工建设，广清一体化步伐不断加快，清远有望步入新一轮较快发展时期。与此同时，国际贸易保护抬头，发达国家“再工业化”，区域经济竞争更为激烈，经济发展不稳定、不确定因素增多，经济下行压力仍然较大。面对新起点、新机遇、新挑战，我们要认清形势，把握机遇，开拓创新，努力开创清远振兴发展新局面。

新一届政府工作目标任务是：经济保持中高速增长，生产总值年均增长8%左右，固定资产投资年均增长12%，地方一般公共预算收入年均增长8%，全社会消费品零售总额年均增长9%；创新驱动水平明显提升，先进制造业和现代服务业比重持续提高，农业现代化取得实质进展；新型城镇化建设稳步推进，“一心两核”辐射带动能力显著提升，常住人口城镇化率超过60%；基本公共服务均等化实现“两个90%”，城乡居民人均年收入增长快于经济增长，力争超过全国平均水平。

新一届政府工作的总体要求是：高举中国特色社会主义伟大旗帜，以邓小平理论、“三个代表”重要思想和科学发展观为指导，深入学习贯彻习近平总书记系列讲话精神，统筹推进“五位一体”总体布局和协调推进“四个全面”战略布局，坚持稳中求进工作总基调，牢固树立和贯彻落实“五大发展理念”，适应把握引领经济发展新常态，坚持以提高发展质量和效益为中心，坚持以推进供给侧结构性改革为主线，贯彻落实市第七次党代会的决策部署，立足“南融北拓桥头堡，水秀山青后花园”战略定位，深入实施广清一体化发展战略，扎实推进“南部地区加快融入珠三角、北部地区全面建成小康社会”两个行动计划，大力振兴实体经济，确保如期全面建成小康社会。

围绕上述目标任务和总体要求，必须着力推进以下重点工作：

（一）促进广清一体化新发展。积极对接融入珠三角，力争在交通、产业、政策方面率先实现与珠三角一体化。积极参与大珠三角经济区建设，深化与珠三角地区的产业共建。深入实施广清一体化发展战略，推动广清两地交通互联、产业共建、城市融合、生态共治、民生共享。加快连接广清两市的高速公路和轨道交通建设，全面贯通两市断头路，促进航道网和港口群无缝对接。推进广清交界产业集聚区建设，重点打造广清产业园清城片区、佛冈片区、广清旅游集聚区和空港经济区。广清产业园力争实现工业增加值400亿元以上。积极承接广州非中心城市功能，引导物流枢纽、商业服务、农批市场等城市功能向我市转移。推动公共服务共建共享，生态环境共保共治。扎实推进北部地区全面建成小康社会行动计划，抓好连阳四县（市）国家重点生态功能区建设，推动生态优势转化为发展优势。做好北部地区交通基础设施发展规划，完善北部地区交通网络。用好中央、省扶持民族地区发展政策措施，加快民族地区全面建成小康社会步伐。

（二）推动交通建设新突破。将交通建设作为加快清远发展的关键所在。着力构建“六纵四横、一江四轨”的综合交通网络。确保佛清从、汕湛、汕昆高速清远段、广清与清连高速连接线建成通车，实现8条高速公路双向56个车道对接珠三角。谋划推动英德、连州通用机场和韶贺高速、磁浮轨道建设。谋划高铁清远站扩容升级，启动“五位一体”交通枢纽建设。加快推进广清城轨延长线及二期建设。完成北江航道扩能升级建设。稳步提高普通国省道和县乡公路通行能力，增强国省道对县城、中心镇、重要旅游区和主要交通枢纽的连接和覆盖。

（三）构建现代产业新体系。大力推动“大众创业、万众创新”，打造创新型城市和粤北区域科技创新中心。将清远国家级高新区打造成为全市创新发展的新“引擎”。高新技术企业力争达到300家以上。加快产业园区提质增效，大力发展实体经济。重点打造汽车零配件、新材料、装备制造、生物医药、电子信息、食品饮料、智能家居等7大产业集群，培育10家左右产值超50亿元的新材料龙头企业，力争形成1-2个产值超500亿元的产业集群。培育发展现代物流、科技服务、文化创意、大数据、农村电商、跨境电商等新业态。依托长隆项目，推进全域旅游、特色旅游，推进国际化旅游城市建设。大力推进农业供给侧结构性改革，提升农业产业化、规模化、效益化水平。改造提升陶瓷、水泥、有色金属等传统产业。加快省级职教基地建设，大力引进培养创新和高技能人才，建设南方职教高地和粤北人才高地。

（四）打造城乡建设新面貌。大力推进城市扩容提质，完善燕湖新城配套设施，加快英德、连州和佛冈副中心城市建设，有序推进县城和中心镇、特色小镇规划建设，加大智慧城市、海绵城市、低碳城市建设力度，推动产业、人口与城市协调发展，提升城市建设水平。继续推进“五城同创”。大力改造“城中村”。加强信息网络基础设施建设，力争在粤东西北率先实现与珠三角一体化。创建“宽带中国”示范城市。编制全域建设规划，实现镇村规划全覆盖。推进镇村道路交通建设，加强集贸市场、公交站场、垃圾处置场等公共设施建设。继续推进美丽乡村、社会主义新农村连片示范建设和名镇名村创建活动。

（五）增创生态环保新优势。扎实推进主体功能区建设，建立健全绿色发展指标体系、资源环境承载能力监测预警指标体系和绩效考核评价体系，构建绿色金融生态圈。加强城市修补、生态修复，更新城市发展模式和治理方式，促进城市与自然和谐发展。加强大气污染综合防治，加快现有工业企业燃煤设施清洁能源替代，全面整治燃煤小锅炉，积极推行清洁生产，加快黄标车及老旧车淘汰进程。加强北江流域保护治理。全面落实“河长制”。加大污水处理厂及管网配套建设力度，确保全市城镇生活污水集中处理率达到90%以上。加强船舶污染控制，做好农业面源污染治理。加强饮用水源保护，确保城市和县级集中饮用水源水质全部达到Ⅲ类以上。加大造林绿化力度，稳步提升森林覆盖率。加强土壤重金属污染修复。建立碳排放权交易制度。

（六）力促改革民生新进展。深化“放管服”改革，加强政府系统廉政建设和干部队伍作风建设，营造法治化便利化营商环境。加快法治政府建设。深化农村综合改革，激发“三农”发展活力。抓好供给侧结构性改革，深入推进“三去一降一补”，推进农业供给侧改革，促进房地产平稳健康发展。创新投融资平台和方式。牢固树立共享发展理念，推进公共服务常住人口全覆盖，持续提升教育、医疗卫生、文化体育等基本公共服务均等化水平，使人民群众更有获得感。加快“四馆三中心”建设。积极申办广东省第16届运动会。扩大和促进就业。持续提高社会保险覆盖面和保障水平。高质量完成精准扶贫脱贫攻坚工程，抓好国家级扶贫改革试验区建设。

2017年工作安排

2017年是新一届政府的开局之年，也是我市转型攻坚的关键一年，做好今年的政府工作意义重大、影响深远。我们将全面贯彻落实市第七次党代会决策部署，紧紧围绕“1112”总体布局，深化供给侧结构性改革，大力振兴实体经济，加大重点项目重大平台建设力度，扎实做好财税金融外经贸工作，着力营造良好的营商环境，力促全市经济社会稳步健康发展，为全面建成小康社会打下坚实基础。

今年经济社会发展的主要预期目标是：生产总值增长8%左右；固定资产投资增长10%以上；一般预算收入可比口径增长8%左右；社会消费品零售总额增长10%；城乡居民可支配收入与经济增长同步；城镇登记失业率控制在3.5%以内，居民消费价格指数预期103.5；全面建成小康社会指数达到88%，社会保障、资源环境保护、节能减排降碳及安全生产完成省定目标。

实现上述目标，必须全力抓好以下十方面工作：

（一）促进区域协调发展。全面实施广清一体化战略。着力解决广清产业园用地问题，力争年内动工企业60家，试投产企业25家。完成广清产业园扩园工作。加快广清产业园佛冈片区开发建设。理顺帮扶区（县）共建园区运营管理机制，尽快进入实质性共建阶段。围绕重要平台，做好规划衔接和路网完善，加快推进广清大道及东环快速路南延线项目。与花都区共建绿色金融改革创新试验区。加快广州北江引水工程项目建设。健全两地环境保护和区域突发环境事件应急协调处理机制。加快供穗“菜篮子”基地建设。积极参与广佛肇清云韶经济圈建设，促进经济圈路网对接联通、产业错位发展。积极融入“一带一路”战略平台，拓宽开放合作空间。加强与高铁沿线城市合作交流。加大对民族地区支持力度，推进连阳四县（市）国家重点生态功能区建设，深化民族工业园合作共建机制，培育壮大一批特色农林业、生态文化旅游产品，加快风电、光伏发电等清洁能源项目建设。推进德建水库、国道323线连阳段改造等项目加快建设，启动连州、英德通用机场规划建设，加快推进柳韶铁路前期工作，争取韶贺高速纳入省重点交通项目建设“笼子”。

（二）加大交通建设力度。加快广清城轨建设，确保一期工程年内建成，争取延长线（延伸至高铁站）开工建设。加快“五位一体”交通枢纽规划建设。积极推进城市出入口环境改造优化。推动佛清从高速、汕湛高速、汕昆高速、“高接高”等高速公路项目加快建设，动工建设广连高速清远段、二广高速与清连高速连州连接线、二广高速连山至贺州支线，力争完成高速公路建设投资100亿元。动工建设清西一级公路。完成国道107线与广清城轨并行（上跨）段路面环境工程。规划实施国道106线佛冈县城段、省道114线广清产业大道路段、省道347线青塘至望埠段等国省道升级改造项目。完成全市362.5公里县道烂路改造，消灭市域内县道烂路。强力抓好治超工作。启动1300公里国省道公路安全生命防护工程。加快推进北江航道扩能升级工程，力争完成投资23.6亿元。完成清远港总体规划修编工作。积极推进大宗货物北江绿色运输示范项目建设。

（三）推进产业转型升级。继续完善产业园区配套建设，加大园区项目用地保障，推动产业园区提质增效，力争全年园区完成工业项目固定资产投资117亿元，规上工业增加值占全市比重30%以上。力争国家级高新区综合排名争先进位。争取成为国家高性能金属材料产业化基地。大力实施“一区多园”管理，推动园区联动发展。针对重点区域重点领域，组织筹备省属企业、广州、深圳等招商对接会，积极争取省属国有企业新增工业项目或生产基地落户我市；针对珠三角地区大型骨干企业和特色优势企业的加工制造环节、增资扩产及转型升级项目，主导产业的配套企业，拥有较稳定国际市场份额的劳动密集型加工贸易企业，规模以上生产性服务业，开展重点招商，争取珠三角更多优质企业向我市转移，确保完成省定任务。加强全市招商引资工作统筹，引导项目科学布局，推进产业集群发展。加大企业帮扶力度，促进各项惠企政策落地。加大企业技改创新扶持力度，推动50家以上规上企业开展技术改造，力争10家以上企业开展机器人应用，新成立2家以上企业工业设计中心。开展水泥行业碳排放权交易试点。鼓励再生资源行业整合。培育高新技术企业，确保年底高新技术企业达120家以上。成立市创投服务中心。设立政府引导的创业投资基金或产业引导基金。加快天安智谷、华南863等创新创业平台建设。推进研发机构建设，力争组建新型研发机构1家，产业技术创新战略联盟1个，孵化器、众创空间6家以上，市级以上工程技术研发中心突破100家。加大力度支持企业上市，力争1-2家企业上IPO，5家企业上“新三板”，30家企业在区域股权交易中心挂牌。加快清新、英德、佛冈等县级中小企业服务中心建设。修订完善上市奖励扶持政策。深入实施质量强市和先进标准体系建设，推动英德红茶、连山大米创建国家或省级知名品牌示范区。进一步优化和落实各项人才政策，积极与高校、科研单位联合培养人才，多渠道引进高素质人才。协助企业解决招工难问题。支持人力资源企业发展壮大。做好土地总体规划中期评估调整工作，完善土地管理共同责任制，依法加大闲置用地处置力度。安排省、市重点项目214项，年度投资410亿元。

（四）加速发展服务产业。加快推进长隆项目建设，启动二期迎咀组团征地拆迁工作，及时做好土规调整和用地报批供应，加快清晖路南延段、平安路、森林大道等路网建设，做好水库功能调整、项目发债等工作。开展旅游集聚区招商推介工作。开发足球、温泉、民俗等特色旅游小镇。推进佛冈、连南、连山、阳山全域旅游示范区创建工作。积极创建全域旅游示范市。加强与广州、深圳、港澳旅游合作与宣传，深耕珠三角客源地和高铁沿线旅游市场。推进北江旅游码头建设。加快自驾车营地、自驾游驿站、标牌标识、服务中心、汽车旅馆等配套建设，扶持自驾旅游发展。启动清远市智慧旅游指挥调度平台建设。支持发展二手车交易市场，大力挖掘汽车消费潜能。引导万达广场、沃尔玛、时代广场等商业综合体发展体验式服务。争取新引进2家以上金融机构，稳妥发展互联网金融。完善农村电商县级运营网络，加强与苏宁、阿里、京东、中国邮政的合作，不断扩大农村电商覆盖面。加强与电信运营商合作，提高信息网络基础设施建设水平。推进电商产业园建设，完善电商产业园服务配套，吸引更多电商经营户入驻。推进源潭物流园、华南声谷等专业园区加快规划建设。

（五）提升城市发展水平。全面开展创文、创卫活动，力争创建成为国家卫生城市、全国文明城市。加快湖城大道、清晖路、松苏岭公园等在建项目建设，推动四大馆动工建设，新启动东环快速南段、峡山西路等一批项目。加快北江四桥建设，确保五桥建成通车。推动省级职教基地3所高职院校动工建设。加快推进江南水厂、滨江备用水源、220千伏岭塘等供水供电项目建设。加强城市地下综合管廊建设。推进城市管理和执法体制改革，升级数字城管平台，提高城市管理水平。进一步完善停车设施。新建一批公交候车亭，开通“清远出行易”APP，加大公交运力投入，优化公交线网。提升城市绿道管养水平。加快市区南、北组团式垃圾综合处理基地项目前期工作，力争上半年动工建设。推进垃圾分类减量和一般镇区垃圾费征收。完成市区“三规合一”和全市旅游特色小镇总体规划编制，指导县市加快“三规合一”编制。推进佛冈、连州省级新型城镇化“2511”试点建设。

（六）推进农业供给侧改革。抓好农综改各项工作落实。积极稳妥推进土地整合。做好涉农资金整合试点工作，建立健全涉农资金整合优化制度。加快农村综合服务平台建设，确保年内建成100个以上。推进农村产权交易管理平台建设，引导鼓励农民和农村产权所有者入市交易。进一步完善农村信用体系建设，稳步推进农村“两权”抵押贷款和普惠金融“村村通”工作。健全农业产业保险体系，开发2-3个农业保险新品种，力争完成巨灾保险试点工作。建设一批村村通自来水工程。完成124.6公里中小河流治理。完成208个中心村农网升级改造。加快农村光纤网络建设。加快推进高标准基本农田项目建设。扎实开展耕地质量保护提升工作。大力培育和发展各类农民专业合作社，提高农民在产前、产中、产后等各个环节的组织化程度，推动农业产业化、规模化、效益化发展。加强“三品一标”农产品监管，推动绿色生产和品牌化建设，争取创建省名牌农产品4个、绿色食品4个、无公害农产品10个、农产品地理标志1个。加快美丽乡村建设，确保年内完成100条以上示范村创建。加强农村人居环境整治。推进省级新农村连片示范项目建设。出台促进乡村旅游发展实施意见，大力发展农家乐、休闲观光农业等，增加村集体和农民收入。

（七）全面加强环境治理。全力冲刺国家环保模范城市创建工作。继续大力整治工业污染、机动车尾气、燃煤污染和工地、道路扬尘、农业面源等污染源，全面落实各县（市）建成区黄标车限行，淘汰禁燃区高污染燃料锅炉。继续优化环境空气自动监测布点，确保空气质量全面稳定达标并持续向好。加快气源工程和天然气管道项目建设，确保天然气管道铺设覆盖禁燃区范围，推动燃气锅炉应用。完成27家省级重点监管企业VOCs整治。认真组织实施《清远市饮用水源水质保护条例》。完成清新与旧城污水处理厂二期工程，加快镇村污水处理设施建设。积极开展市、县、镇三级集中式饮用水源地规范化建设。加快黄坑河、澜水河、海仔大排坑、龙沥大排坑等4条黑臭水体综合整治。逐步提升大燕河、乐排河、笔架河、潖江、滨江、漫水河等河流水质。推进龙塘、石角电子废弃物污染场地及土壤修复示范工程建设，完成遗留残渣清运、阻隔和围蔽。

（八）大力发展民生事业。全面落实精准帮扶措施，继续深化扶贫品牌建设，重点抓好产业扶贫、技能培训和劳务输出、社会扶贫、金融扶贫，力争全市261个贫困村土地整合实现全覆盖；认真抓好督导工作，落实各级责任，确保完成年度4.2万贫困人口的脱贫任务。全力推进住房保障工作，确保完成省定任务。深化就业创业体制机制改革，支持高校毕业生、外出务工人员返乡创业，实现全市城镇新增就业4万人。依法推动企业全员足额参保，实现基本社会保险覆盖率98%以上。探索推进城镇职工和城乡居民养老保险一体化。推进社会养老服务体系建设，抓好公办养老机构社会化改革试点。加快机关事业单位养老保险制度改革，同步建立职业年金制度。大力发展慈善事业。推进教育“创先”工作，确保清城、佛冈、连南“创先”成功，加大中心城区、县城学位和优质学位供给，大力推进民办义务教育学校标准化建设。开展卫生强市建设，深化公立医院改革，有序推进中心卫生院改造升级为县级综合医院，继续推进乡镇卫生院标准化建设，促进医疗资源向基层下沉，启动分级诊疗建设试点，确保家庭医生签约服务覆盖率达15%以上。全面落实两孩政策，着力提升妇幼健康服务水平。继续开展好清远马拉松、广场文化艺术节等群众性文体活动。加快基层三防能力建设。推进食品安全快速检测站和乡镇农产品质量安全检测体系建设。切实抓好安全生产。扎实推进“平安清远”建设和社会治理创新。抓好打假、打传、打私工作。

（九）深化重点领域改革。推进“去降补”各项任务落实，加快完成国有“僵尸企业”出清重组，力争实现2家特困企业市场出清，实现13家以上困难企业脱困；积极推动房地产去库存，引导农村人口有序进城购房，提高棚改货币化安置比例，推动住房租赁平台组建和运营，促进房地产业健康稳定发展；提高金融风险预警和防控能力，严控高杠杆、高风险融资项目，严厉打击非法集资行为；落实好国家和省确定的各项降成本政策措施，推进我市大用户直购电和售电侧改革，推进营改增全面扩围，取消减免一批政府性基金，扩大小微企业免征范围，降低企业生产经营成本。抓好相对集中行政许可权试点，建设大监管平台，开展政务服务大数据建设，设立清远高新区行政审批局，完善“一门式一网式”政府服务模式。抓好企业登记多证合一试点。逐步推进登记注册全程电子化。继续清理规范政府部门行政审批中介服务事项，建立中介管理平台。继续做好行政许可实施和监管情况评价。实施“双随机、一公开”监管全覆盖。推进综合行政执法改革，探索镇（街）一支队伍执法。配合开展省级以下环保机构检测监察执法垂直管理改革。加快推进承担行政职能事业单位改革试点。公布第二批事业单位公共服务事项清单。

（十）加强政府自身建设。全市政府系统各级各部门必须深入学习贯彻习近平总书记系列重要讲话精神，切实增强政治意识、大局意识、核心意识、看齐意识，特别是核心意识、看齐意识，按照“三严三实”的要求，自觉接受人大及其常委会依法监督和人民政协民主监督，严格执行各项决议决定，定期向市人大及其常委会报告工作和向市政协通报情况，办好人大代表建议和政协委员提案。充分发挥政府顾问、法律顾问作用，认真听取民主党派、工商联、无党派人士和各人民团体意见建议，提高决策科学化、民主化水平。坚持依法行政，深入推进法治政府建设。继续落实行政首长出庭应诉工作。进一步提升12345政府服务热线服务效能。持续推进机关作风建设，深入开展“正风”行动、“公述民评”等活动，大力治理“庸懒散奢”和“为官不为”问题。建立“亲”“清”新型政商关系，营造干事创业的良好氛围，努力建设为民廉洁高效政府。加强统计、民族、宗教、外事、侨务、港澳、对台、人防、国防动员、档案、方志、气象、水文、慈善、妇女儿童、残疾人等工作。

各位代表，在落实省十件民生实事各项任务的同时，今年市政府将投入3.86亿元，全力办好十件民生实事：一是继续提升底线民生保障水平；二是全面完成4.2万人年度脱贫任务；三是提高教育发展保障水平；四是提升基层医疗卫生服务水平；五是开展创业就业万人培训活动；六是加快农村困难户危房改造；七是推进农村公路和危桥改造建设；八是实施城中村污水处理管网改造建设；九是扩大农业种养保险补贴；十是全市推进100条内街小巷改造。

各位代表，未来五年是清远实现跨越发展、全面建成小康社会的决胜期，做好2017年各项政府工作意义重大、使命光荣。让我们更加紧密团结在以习近平总书记为核心的党中央周围，按照省委、省政府和市委的决策部署，解放思想、坚定信心、锐意进取，为清远加快振兴发展、全面建成小康社会而努力奋斗！
